# Phytochemical content of *Cycas rumphii n*-butanol fraction and antiprotozoal activity against *Toxoplasma gondii in vivo*

**DOI:** 10.1038/s41598-025-98993-y

**Published:** 2025-05-05

**Authors:** Hosam M. El-Seadawy, Amany E. Ragab, Mona El-Aasr, Kamilia A. Abo El-Seoud, Ayat A. Elblihy, El-Sayed El-Alfy, Hanan Abd Elgawad, Somaya Saleh, Heba Sheta, Rana Elseadawy

**Affiliations:** 1 Department of Pharmacognosy, Faculty of Pharmacy, Tanta University, Tanta, 31527 Egypt; 2 Department of Medical Parasitology, Faculty of Medicine, Mansoura University, Mansoura, 35516 Egypt; 3 Program of medicine and surgery, Mansoura National University, Gamasa, 35516 Egypt; 4 Parasitology Department, Faculty of Veterinary Medicine, Mansoura University, Mansoura, 35516 Egypt; 5 Department of Pathology, Faculty of Medicine, Mansoura University, Mansoura, 35516 Egypt

**Keywords:** *T. gondii*, *C. rumphii*, *n*-butanol, Phenolics, ME49 strain, HPLC, Drug discovery, Microbiology

## Abstract

Toxoplasmosis treatments are currently limited due to their ineffectiveness against the chronic stage and potential adverse effects. The search for novel medications that possess characteristics such as non-toxicity, and efficacy on all stages of the parasite, particularly the cystic form, is important. The *n*-butanol (*n*-BuOH) fraction of *Cycas rumphii* has shown promising potency against *Toxoplasma gondii* (RH strain) in vitro, and this study aimed to evaluate its efficacy against *T. gondii* in mice models. Therefore, mice were infected with 20 cysts (acute infection model) or 10 cysts (chronic infection model) of *T. gondii* (ME49 strain). The infected mice were treated with *C. rumphii n*-BuOH fraction and cotrimoxazole at 200 mg/kg/day and 370 mg/kg/day, respectively, divided into 2 doses/12 h, starting the 4th day (acute stage) or the 6th week (chronic model) post-infection, and continued for 14 consecutive days. Mice were sacrificed at 56, and 70 days post-infection for acute and chronic models, respectively, to assess the efficacy of treatment. Evaluations included size and count of brain cysts, histopathological examinations of the brain and liver, inflammatory scoring, and immunological studies to assess the levels of nitric oxide (NO), interferon-gamma (INF-γ), and tumor necrosis factor-alpha (TNF-α) in serum. Oral administration of the *n*-BuOH fraction at a daily dose of 200 mg/kg for two weeks significantly reduced the cyst count in the brains of infected mice and improved the survival rate. The results were comparable to those observed in subgroups treated with cotrimoxazole in both acute and chronic models. The histopathological findings indicated that *C. rumphii n*-BuOH alleviated *T. gondii*-induced brain and liver lesions. Furthermore, the serum levels of NO, and IFN-γ were significantly elevated in the *C. rumphii n*-BuOH fraction-treated subgroups. High-performance liquid chromatography analysis indicated that this fraction is rich in phenolic acids, including caffeic acid, gallic acid, and chlorogenic acid which have been linked to anti-oxidant, anti-inflammatory, antiparasitic, and anti-*Toxoplasma* properties. Experimental results demonstrated that the *n*-BuOH fraction of *C. rumphii* exhibits anti-*Toxoplasma* effects, making it a promising candidate for the development of complementary therapies or use in combination with other drugs.

**Supplementary Information:**

The online version contains supplementary material available at 10.1038/s41598-025-98993-y.

## Introduction

*Toxoplasma gondii*is one of the most common parasites that infect humans and other warm-blooded animals with a global distribution^1–3^. Cats serve as the definitive hosts for *T. gondii*, while humans and other intermediate hosts can become infected through various routes. Infection can occur by ingestion of oocysts from the environment, or contaminated fruits, vegetables, and water. The most likely source of infection through food is the consumption of raw or undercooked meat contaminated with tissue cysts. Additionally, transmission can occur by tachyzoites through consumption of raw milk, or transplacental transmission^1,4–6^. Infections in healthy individuals, primarily asymptomatic while, in immunocompromised patients such as those with HIV/AIDS or undergoing organ transplantation, can develop encephalitis^7,8^. Congenital infection occurs during pregnancy, and can lead to clinical signs ranging from asymptomatic cases to severe brain and ocular involvement, and abortion^4,9^. On the contrary, abortions are the most significant clinical manifestation in farm animals, particularly in sheep, and they can lead to substantial economic losses^10,11^.

Effective therapeutic treatments for toxoplasmosis are currently limited, with no ideal treatment for the chronic stage of the infection due to poor brain penetration and potential adverse effects^12–14^. The most commonly used therapy for toxoplasmosis is the combination of pyrimethamine and sulfadiazine, which targets the acute form of the infection, reducing parasite multiplication in the early stages^14,15^. However, this combination has been associated with a number of adverse effects, including neutropenia, low platelet count, thrombocytopenia, leukopenia, and hypersensitivity reactions^16^. Other treatments have been used, such as combining pyrimethamine with clindamycin, atovaquone, clarithromycin, azithromycin or trimethoprim‑sulfamethoxazole but they have been found also less effective against the latent stage of the infection and can be associated with side effects^13,15,17^.

The search for novel medications with characteristics such as placental penetration, non-toxicity, and efficacy on all stages of the parasite, particularly the cystic form, is important. Thus, studies have been conducted on medicinal plant extracts and their natural metabolites as promising alternative safe therapeutics for toxoplasmosis^18–20^. *Cycas rumphii* Miq., commonly known as queen sago or false sago, is a palm-like tree belonging to the Cycadaceae family, which is one of the largest families of gymnosperms. This family consists of approximately 120 species, all within the single genus *Cycas*^21^. *C. rumphii *is characterized by its large, hard, and glossy leaflets that have broad bases, and a long petiole that is almost armed^22^. This species is primarily found on the Moluccan islands of Indonesia^23^. In Southeast Asian cultures, *C. rumphii *is used for various purposes, including as an aphrodisiac, narcotic, and stimulant, as well as for topical applications to heal ulcers, wounds, and skin lesions^24^. Extracts from *C. rumphii *have demonstrated effective antibacterial and anticancer properties^25–27^. However, the antiparasitic properties of various *Cycas* species have not been thoroughly investigated. Notably, the *n*-BuOH fraction of *C. rumphii* has shown promising potency against *T. gondii*(RH strain) in vitro, when compared to the standard drug cotrimoxazole^27^. Nevertheless, further investigation of this potent fraction in animal models is required. This study was designated to evaluate the efficacy of the *C. rumphii n*-BuOH fraction against both acute and chronic *T. gondii* infection (ME49 strain) in mice models. In addition, analysis of the phytochemical content of this fraction using high-performance liquid chromatography (HPLC) was conducted to identify and quantify the major flavonoids and phenolic contents that may contribute to its activity.

## Materials and methods

### Preparing *n*-BuOH fraction of plant material

*Cycas rumphii* leaves were gathered in July 2018 from El-Abd Garden along the Cairo-Alexandria desert road. It was kindly provided and identified by Rabea Sharawy, agronomist and palm researcher. A voucher sample (No. PGG-012) was stored in the herbarium at Department of Pharmacognosy, Faculty of Pharmacy, Tanta University, Egypt. The plant leaves were shade-dried, pulverized into a powder, and stored in carefully sealed containers. Five kilograms of the plant powder were extracted using methanol through a cold maceration process until the extraction was completed. The methanol extract was then evaporated at 40 °C under reduced pressure, resulting in a green residue weighing 294 g. This residue (294 g) was fractionated into four different fractions using petroleum ether, methylene chloride, ethyl acetate, and *n*-butanol (*n*-BuOH). The yields for these fractions were 38.03 g, 8.10 g, 20.1 g, and 59.20 g, respectively. An HPLC analysis of 300 mg of the *n*-BuOH fraction was performed.

### Phytochemical analysis

#### HPLC analysis of *C. rumphii n*-BuOH fraction

HPLC analysis of the *n*-BuOH fraction from *C. rumphii* was conducted using an Agilent 1260 series apparatus equipped with a Zorbax Eclipse Plus C18 column (4.6 mm × 250 mm internal diameter, 5 μm). The mobile phase consisted of water (A) and 0.05% trifluoroacetic acid in acetonitrile (B). The flow rate for separation was set at 0.9 mL/min. A linear gradient was programmed for the mobile phase as follows: at 0 min (82% A), from 0 to 1 min. (82% A), from 1 to 11 min. (75% A), from 11 to 18 min. (60% A), from 18 to 22 min. (82% A), and from 22 to 24 min. (82% A). A multi-wavelength UV detector was used to monitor the analysis at 280 nm. The column temperature was maintained at 40 °C. Sample solutions were injected in volumes of 5 µL. Prior to injection, each sample was filtered using an Acrodisc syringe filter (Gelman Laboratory, MI, USA) with a pore size of 0.45 μm. Peaks were identified and compared to standards based on UV spectra and consistent retention times.

The investigation utilized 18 analytical standards supplied by Merck^®^ (New Jersey, USA), which can be categorized into two groups: simple phenols/phenolic acids and flavonoids.

#### Determination of total phenolic and flavonoid contents

##### Preparation of sample and standard solutions

Standard stock solutions of gallic acid and rutin were prepared at a concentration of 2 mg/mL in methanol. For the determination of total phenolic content, subsequent dilutions of gallic acid were made to achieve concentrations of 1000, 750, 500, 375, 250, and 187.5 µg/mL. For total flavonoid determination, the rutin dilutions included concentrations of 1000, 500, 250, 125, 62.5, and 31.25 µg/mL. The *C. rumphii n*-BuOH sample was extracted in ethanol at a concentration of 5 mg/mL.

##### Total phenolic content (TPC) determination procedures

The total phenolic content (TPC) was determined using the Folin–Ciocalteu method^28^. In a 96-well microplate, 10 µL of the sample or each diluted standard concentration was combined with 100 µL of Folin-Ciocalteu reagent (diluted 1:10). Then, 80 µL of 1 M Na_2_CO_3_ was added to the mixture, and incubated in the dark for 20 min at room temperature (25 °C). After the incubation period, the resulting blue complex was measured at a wavelength of 630 nm. The average readings from three replicates were calculated, and a standard calibration curve along with a regression equation was created based on the mean absorbance of various concentrations of gallic acid (**Table ****S1** and **Figure ****S1****A**). The TPC of *C. rumphii n*-BuOH extract was calculated as gallic acid equivalents (µg gallic acid eq/mg sample). Means ± standard deviation (SD) are used to represent data.

##### Total flavonoid content (TFC) determination procedures

The total flavonoid content (TFC) was determined using the aluminum chloride method^29^. In a 96-well microplate, 15 µL of the sample or standard was added, followed by 175 µL of methanol and 30 µL of a 1.25% aluminum chloride (AlCl_3_) solution. Next, 30 µL of 0.125 M sodium acetate was added, and the mixture was incubated for five minutes. After the incubation period, the resultant yellow color was measured at 420 nm. The average readings from three replicates were calculated, and a standard calibration curve along with a regression equation was created from the mean absorbance of various concentrations of rutin **(Table S2**,** Figure ****S1****B)**. The TFC of *C. rumphii n*-BuOH extract was calculated as rutin equivalents (µg rutin eq/mg sample). Means ± SD are used to represent data.

## Parasite and drugs

The avirulent ME49 *T. gondii* strain (type II) was maintained at Parasitology Department laboratory, Faculty of Medicine, Mansoura University, Egypt by passing it through Swiss-albino mice. Brain suspensions were prepared from infected mice eight weeks post-infection by homogenizing the harvested brains in 1 mL of buffered saline (pH 7.2). Light microscopy with a high-power lens (x40) was used to count the number of cysts in the homogenate. Septrin^®^(cotrimoxazole: Trimethoprim‑sulfamethoxazole) oral suspension (GlaxoSmithKline, Egypt) was used as a control drug at a dose of 370 mg/kg/day^30,31^.

Five mice 20–25 g were used in a pilot experiment for selecting the dose of the tested extract. *Cycas rumphii n*-BuOH fraction powder was dissolved in 1% DMSO-PBS and orally administrated to four mice at a dosage of 50, 100, 200, and 400 mg/kg divided in 2 doses/12 h/daily for two weeks. The remaining mouse served as the control and all mice were observed for ten weeks. Mice were evaluated for clinical signs, changes in behaviour, changes in body weight, and food consumption. No mouse mortality has been documented among all mice. Over the ten-week period, no clinical symptoms were observed at dosages of 50, 100 or 200 mg/kg. However, after the 2nd week, there was a gradual decrease in both body weight and food intake, and finally latency to move were observed at dosage of 400 mg/kg but not died. The safe dose for mice without adverse effects (200 mg/kg), was then used to evaluate the efficacy of the tested fraction against *T. gondii* in vivo.

### Experimental animals and design

Thirty female Swiss albino laboratory mice, aged six to eight weeks and weighing between 20 and 25 g, were utilized in this study. The mice were housed individually in separate cages within a well-ventilated room at the Faculty of Medicine, Mansoura University. They were fed a standard commercial diet, and their bedding was replaced daily. Examination of the mice’s stool samples confirmed that they were free of parasitic infections. They were divided into two main groups (I, II) and orally inoculated with *T. gondii *cysts. The first group I (15 mice) was orally inoculated with 20 cysts per mouse, while for the second group II (15 mice) was orally inoculated with 10 cysts^32,33^.

**Fig. 1:**
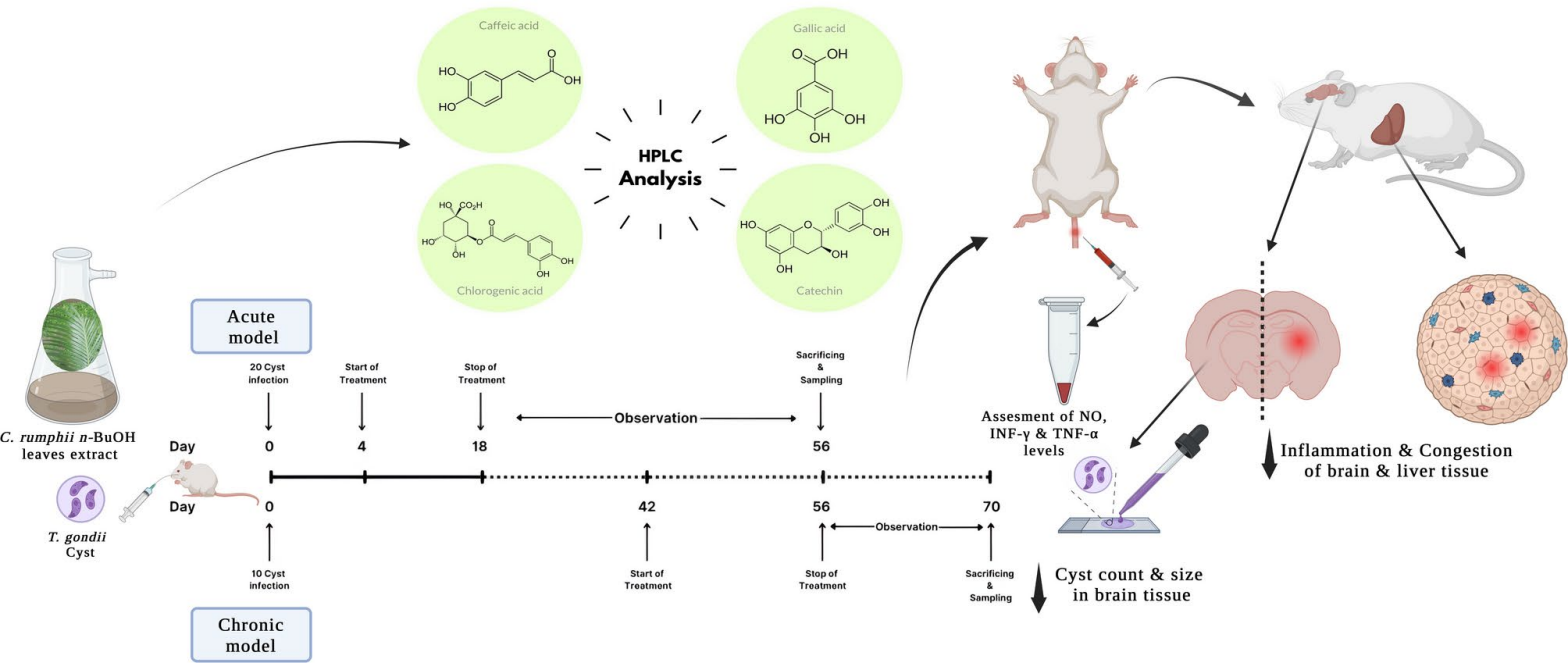
Schematic presentation of the study design. Created in BioRender; Elseadawy, H. (2024), https://BioRender.com/n74o786.

Group I was further subdivided into three groups (five mice per each) as follows: Ia; infected non-treated mice (control group), administrated 1% DMSO-PBS only (vehicle), Ib; infected and treated with the control drug, cotrimoxazole, at a dose of 370 mg/kg/day, and Ic; infected and treated with the tested plant extract (*C. rumphii n*-BuOH fraction) at a dose of 200 mg/kg/day. The tested drugs doses were divided in 2 doses/12 h, started 4 days post-infection (pi) at the acute stage of the infection, and orally administered to mice daily using a stomach tube for 14 days^30^. Mice of all three groups were assessed daily for any clinical signs for five weeks post-treatment, and finally sacrificed at 56 days post-infection (Fig. 1).

Group II was further subdivided into three groups (five mice per each) as follows: IIa; infected non-treated mice (control group), administrated 1% DMSO-PBS only (vehicle), IIb; infected and treated with the control drug, cotrimoxazole, at a dose of 370 mg/kg/day, and IIc; infected and treated with the tested plant extract (*C. rumphii n*-BuOH fraction) at a dose of 200 mg/kg/day. The tested drugs doses were divided into 2 doses/12 h, orally administered to mice daily using a stomach tube for 14 days^30^, started 6 weeks pi at the chronic stage of the infection^34^, and assessed daily for clinical signs for 2 weeks post-treatment^33,35^, and finally sacrificed at 10 weeks post-infection (pi).

At the end of the experimental period, mice were sacrificed through anesthesia with isoflurane by the inhalation route and euthanasia by cervical dislocation.

### Parasitological assessments

The brain from each of the sacrificed mice was collected separately. Half of each brain was rinsed in PBS, weighed, and then homogenized in 1 mL PBS using the Omni TH-220 (GA, USA) for five minutes. After homogenization, 0.1 mL of the homogenate was spread on a clean slide, air dried, fixed in methanol, and stained with Giemsa stain (Merck, Darmstadt, Germany) for 30–45 min. The stained slides were then washed with water, dried, and the total number of cysts was counted and multiplied by 20 to obtain the number of cysts per mouse brain. The mean number of cysts per group was calculated for comparison between the groups of mice. Furthermore, the size of the cysts was measured using an ocular micrometer.

### Histopathological study

The other half of the brain, including grey and white matter of the cerebrum as well as liver of each sacrificed mouse, were fixed in formaldehyde (10% in PBS) at pH 7.2 for subsequent histopathological study using hematoxylin and eosin (H&E) stain. The inflammatory score in the brain was determined under x40 magnification from in five microscopic fields per each mouse^36,37^, as follow: score 0; no inflammation, no necrosis, no gliosis, score 1; mild inflammation, mild gliosis, and scattered giant cells, score 2; moderate inflammation, moderate gliosis, and few giant cells, and score 3; severe inflammation, severe gliosis, and frequent giant cells.

### Measurement of nitric oxide (NO), and Proinflammatory cytokines

Blood samples were obtained from three mice of each subgroup from tail vein before being sacrificed to separate serum. The levels of nitric oxide (NO) in the serum were evaluated using Rat Total Nitric Oxide ELISA Kit (MyBioSource, USA), following the manufacturer’s guidelines. The levels of interferon-gamma (IFN-g), and tumor necrosis factor α (TNF α) were evaluated following the manufacturer’s guidelines for the commercial kits Rat IFN Gamma ELISA Kit, and Rat TNF α ELISA Kit (Sigma-Aldrich, Japan).

### Statistical analysis

Statistical analysis was performed using GraphPad Prism 9.0 (GraphPad Software, San Diego, CA, USA), and a one-way analysis of variance (ANOVA) test was performed followed by Tukey’s multiple comparisons test. The level of significance was shown as asterisks, and a value of *p* < 0.05 was considered statistically significant. Data represent the mean ± SD.

### Ethics declarations

All experimental approaches were conducted according to the ethical principles, guidelines and regulations of Mansoura University, and were approved by Mansoura University Animal Care and Use Committee (MU-ACUC) with approval code number: VM.R.24.08.175. The experiment was performed in accordance with the ARRIVE guidelines.

## Results

### Phytochemical content of *C. rumphii n*-BuOH fraction

**Fig. 2:**
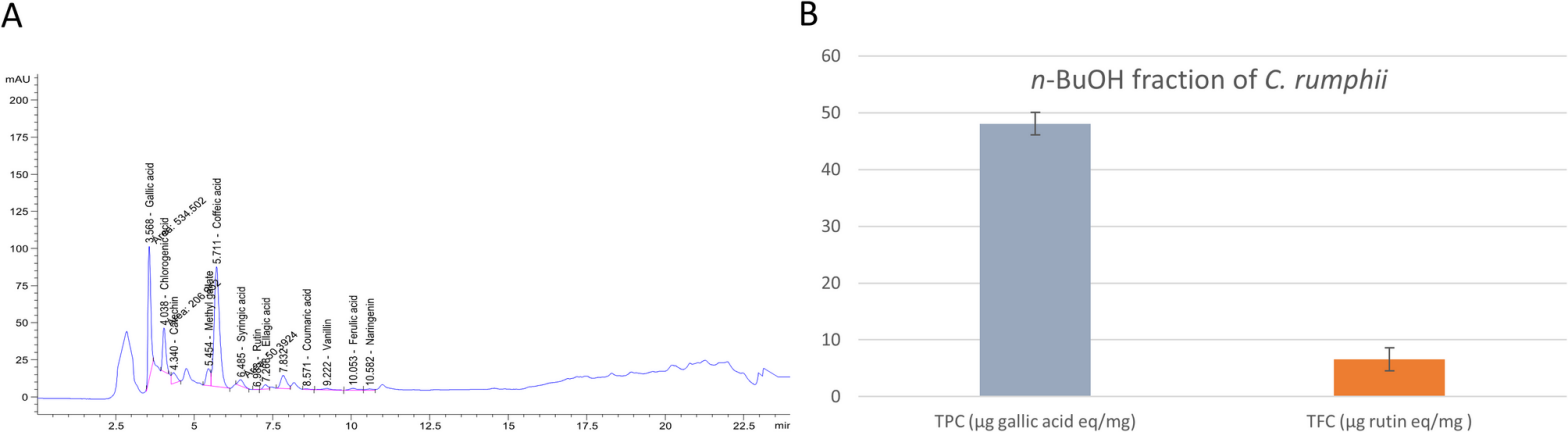
Phytochemical content of *n*-BuOH fraction of *C. rumphii* leaves. **(A)** HPLC/UV chromatogram of *n*-BuOH fraction of *C. rumphii* leaves. **(B)** Comparison between TPC and TFC of *n*-BuOH fraction of *C. rumphii* leaves.

The phenolic and flavonoid compounds included in *C. rumphii n*-BuOH leaves fraction were identified and quantified using HPLC. Twelve phenolic and flavonoid molecules were detected in the HPLC chromatogram of *C. rumphii n*-BuOH leaf extract (Fig. 2A; Table 1). Caffeic acid (293.34 µg/g), gallic acid (143.49 µg/g) and chlorogenic acid (88.05 µg/g) were the main phenolic components. However, the most prevalent flavonoid was (+)-catechin (15.58 µg/g). *C. rumphii n*-BuOH leaves fraction had a total phenolic content of 48.07 ± 2.1 µg gallic acid eq/mg dried plant material. However, the total flavonoid content is 6.56 ± 0.2 µg rutin eq/mg dried plant material **(**Fig. 2B**)**.

**Table 1:** HPLC analysis of phenolic and flavonoid components of *n*-BuOH fraction of *C. rumphii* leaves.

No.	Identified compound	Phytochemical class	RT (Min)	Area (mAU*S)	Area %	Conc. (µg/g)
1	Gallic acid	Phenolic acid	3.56	534.50	24.86	143.49
2	Chlorogenic acid	Cinnamic acid ester	4.03	206.95	9.62	88.05
3	(+)-Catechin	Flavanol	4.34	76.94	3.57	15.58
4	Methyl gallate	Phenolic acid ester	5.45	106.39	4.94	24.32
5	Caffeic acid	Hydroxycinnamic acid	5.71	970.78	45.15	293.34
6	Syringic acid	Phenolic acid	6.48	50.39	2.34	11.59
7	Rutin	Flavonoid glycoside	6.98	8.58	0.03	1.28
8	Ellagic acid	Phenolic acid	7.26	28.59	1.33	8.38
9	Coumaric acid	Hydroxycinnamic acid	8.57	4.12	0.19	0.45
10	Vanillin	Phenolic aldehyde	9.22	17.54	0.81	3.34
11	Ferulic acid	Hydroxycinnamic acid	10.05	23.66	1.10	4.41
12	Naringenin	Flavanone	10.58	14.56	0.67	2.90

### **In vivo efficacy of*C. rumphii n*-BuOH fraction against*T. gondii***

In acute model (Group I), two mice died in the control non-treated subgroup (40%), while one mouse only died from each of Ib and Ic treated subgroups. However, in the chronic group (II) only one mouse died from the non-treated control subgroup (IIa), and none of the treated subgroups (IIb, IIc) died. Mice died suddenly in early weeks after infection without any obvious clinical manifestation. The survived mice were then sacrificed at 56 days and 10 weeks post-infection for acute, and chronic groups, respectively, for parasitological, histopathological, and immunological assessments.

#### *T. gondii* cysts count and size in the brain

**Fig. 3:**
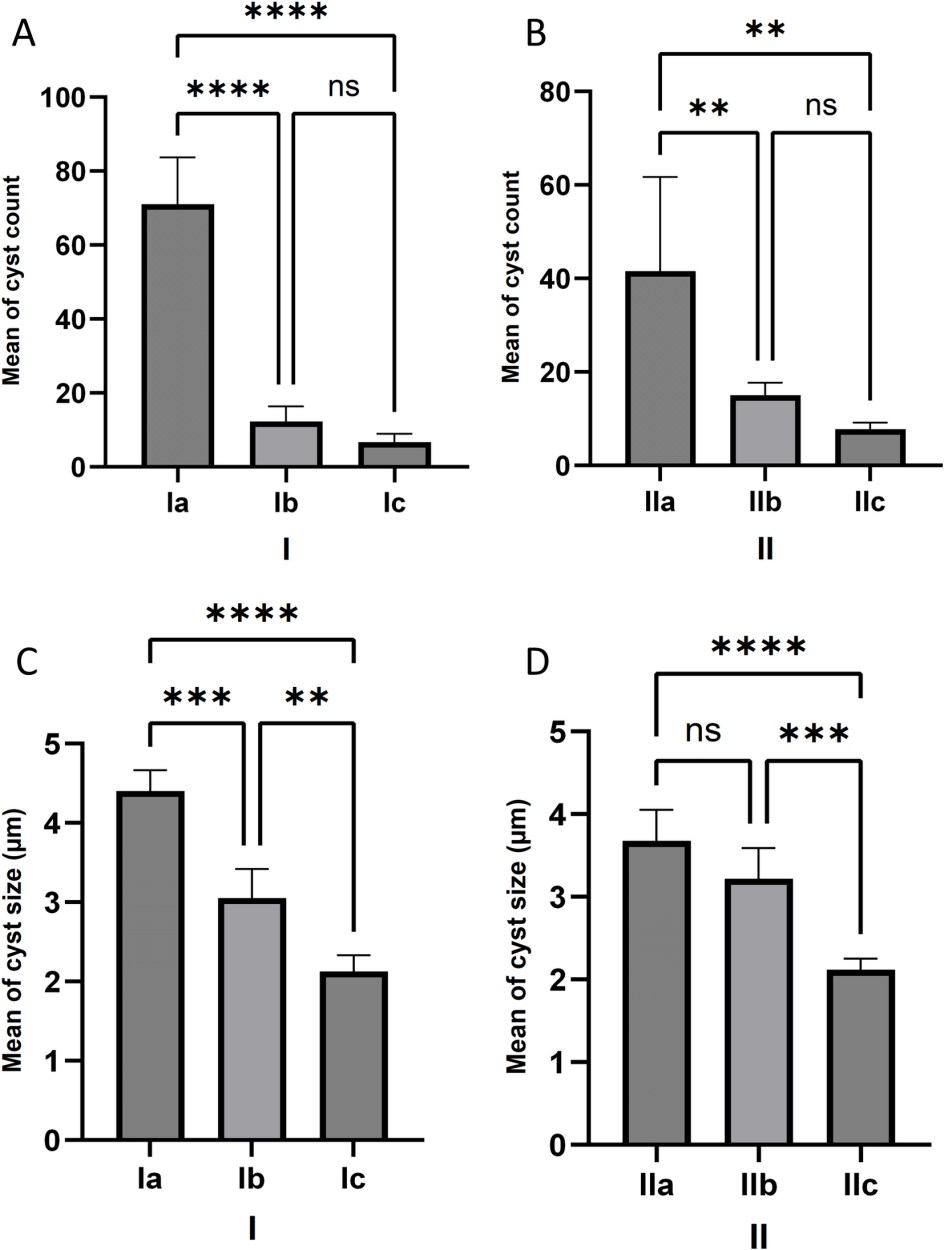
The number (**A** and **B**), and size (**C** and **D**) of *T. gondii* cyst in the brain of experimental subgroups. Results are expressed as mean ± standard deviation and differences were considered significant at *p* < 0.05 (*), *p* < 0.01 (**), *p* < 0.001 (***), and *p* < 0.0001 (****) or non-significant (ns). The first group I: was orally inoculated with 20 cysts per mouse, and the second group II: was orally inoculated with 10 cysts. Ia, IIa: infected non-treated mice (control group), administrated 1% DMSO-PBS only (vehicle); Ib, IIb: infected and treated with the control drug, cotrimoxazole, at a dose of 370 mg/kg/day; and Ic, IIc: infected and treated with the tested plant extract (*C. rumphii n*-BuOH fraction) at a dose of 200 mg/kg/day.

*C. rumphii n-*BuOH fraction treatment resulted in a 90.6% reduction in brain cyst counts in comparison to 82.7% reduction by cotrimoxazole in the acute model, and an 81.4% reduction compared to 63.9% reduction by cotrimoxazole in the chronic model. However, none of the treated mice achieved complete eradication of *T. gondii* cysts. There was a significant reduction in cysts counts in acute treated subgroups (*p* < 0.0001), Ib (12.30 ± 4.07) and Ic (6.67 ± 2.25) in comparison with the non-treated control group (71.03 ± 12.68), while, the difference between cotrimoxazole and *C. rumphii n*-BuOH fraction was non-significant (*p* = 0.51) (Fig. 3A). Likewise, the difference of cysts count was significant in chronic treated subgroups (*p* < 0.01), IIb (15.0 ± 2.70) and IIc (7.74 ± 1.39) in comparison to non-treated control IIa (41.55 ± 20.14), and non-significant between cotrimoxazole and *C. rumphii n*-BuOH fraction treated subgroups (*p* = 0.54) (Fig. 3B). On the other hand, the cyst size of acute treated subgroups (Ib, Ic) was significantly decreased in comparison with the control group (*p* < 0.001 and < 0.0001, respectively) (Fig. 3C). In contrast, the cyst size was significantly decreased in the *C. rumphii n*-BuOH fraction treated chronic subgroup in comparison to non-treated and cotrimoxazole treated chronic subgroups (*p* < 0.0001 and 0.001, respectively), but insignificant between cotrimoxazole and control subgroups (Fig. 3D).

#### Histopathological changes of the brain and liver

**Fig. 4:**
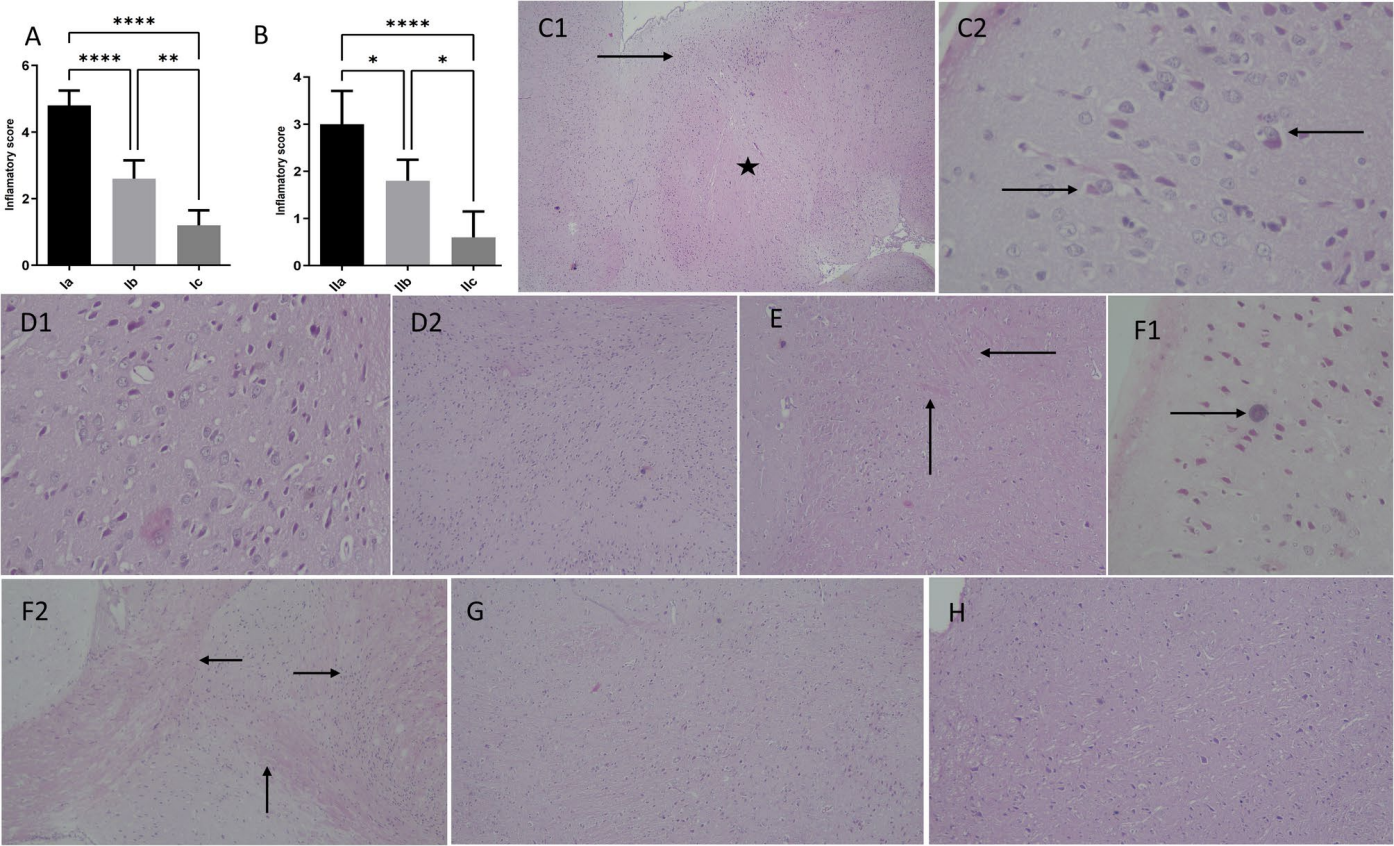
The inflammatory score (**A** and **B**), and histopathological changes (**C**-**H**) in *T. gondii* infected brains of experimental subgroups. (**C**) Acute non-treated: (C1) Shows brain tissue with areas of necrosis and neuronal loss (Asterix) surrounded by marked gliosis and mononuclear inflammatory infiltrate (arrow) (H&E, 4x); (C2) Shows dead red neurons with focal neuronophagia by phagocytic cells (arrows) (H&E, 20x). (**D**) Acutely infected mice treated by cotrimoxazole show some histopathological improvement with areas showing moderate gliosis and inflammatory infiltrate (D1) (H&E, 20x). However, other areas show severe inflammation (D2) (H&E, 10x). (**E**) Acute drug shows mild gliosis and inflammation (arrows) (H&E, 10x). (**F**) chronic non-treated, (F1) shows Toxoplasma cyst (arrow) surrounded by red necrotic neurons (H&E, 20x), (F2) shows moderate to severe gliosis and inflammation (arrows) (H&E, 10x). (**G**) chronic cotrimoxazole group shows mild to moderate inflammatory infiltrate (H&E, 10x). (**H**) chronic drug group shows near normal brain histology (H&E, 10x).

The brains of mice infected with *T. gondii* in the acute non-treated subgroup exhibited areas of necrosis and neuronal loss, with significant gliosis and mononuclear inflammatory infiltrate, resulting in a mean inflammatory score of 4.80 ± 0.44 (Fig. 4A and C). In contrast, acutely infected mice treated with cotrimoxazole showed some histopathological improvement, with regions displaying moderate gliosis and inflammatory infiltrate, although other areas still exhibited severe inflammation (Fig. 4D). The subgroup treated with *C. rumphii n*-BuOH fraction showed mild gliosis and inflammation (Fig. 4E), and had a significant reduction in the inflammatory score (1.20 ± 0.44) compared to the other acutely infected subgroups, Ia (*p* < 0.0001), and Ib (*p* < 0.01). Likewise, the inflammatory score in the non-treated chronic subgroup was significantly higher than in both the cotrimoxazole-treated (*p* < 0.05), and *C. rumphii n*-BuOH fraction treated (*p* < 0.0001) chronic subgroups (Fig. 4B). The chronic non-treated subgroup’s brains displayed cysts surrounded by necrotic neurons, accompanied by moderate to severe gliosis and inflammation (Fig. 4F). In contrast, the cotrimoxazole-treated subgroup demonstrated mild to moderate inflammatory infiltrate and moderate gliosis (Fig. 4G). Meanwhile, the *C. rumphii n*-BuOH fraction treated subgroup exhibited near-normal brain histology (Fig. 4H).

**Fig. 5:**
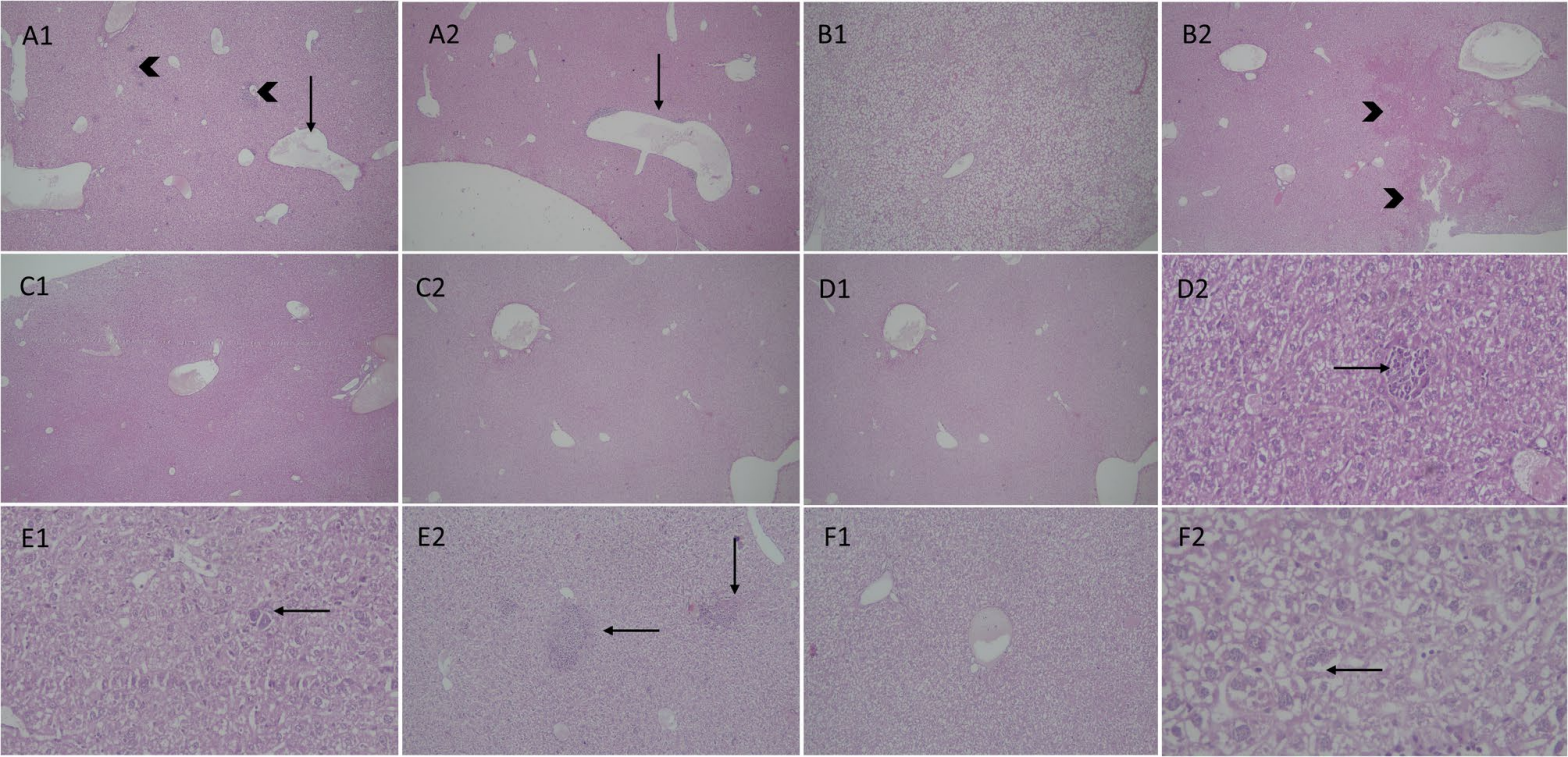
The histopathological changes in liver of experimental subgroups (**A**-**F**). (A1, 2) Acute non-treated group liver shows marked vascular congestion (arrows), pericentral, portal inflammation and focal lytic necrosis (arrow heads) (H&E, 4x). (**B**) cotrimoxazole causes marked deterioration of liver tissue (B1) with marked hepatocyte hydropic degeneration and multiple focal lytic necrosis (H&E. 4x), (B2) Liver tissue with vascular congestion and parenchymal confluent necrosis (arrow heads) (H&E, 4x). (C1, 2) Acute drug group Liver tissue with nearly normal histology (absent congestion, inflammation, necrosis or degeneration) (H&E, 4x). (**D**) Chronic non-treated group liver tissue shows (D1) marked vascular congestion and inflammatory infiltrate and portal mononuclear inflammatory infiltrate (H&E, 10x), (D2) shows focal lytic necrosis (arrow) and marked hepatocyte hydropic degeneration (H&E, 20x). (**E**) Chronic cotrimoxazole liver shows (E1) less vascular congestion and presence of giant cells (arrows) (evidence of repair) (H&E, 20x), (E2) shows prominent focal lytic necrosis (arrows) with less vascular congestion (H&E, 10x). (**F**) Chronic drug liver tissue that (F1) shows mild vascular congestion, absent pericentral, portal inflammation nor focal lytic necrosis (H&E, 10x), (F2) evidence of repair (binucleated hepatocytes) (arrow) (H&E, 40x).

Regarding the histopathological changes in the liver across the experimental subgroups, the acute non-treated subgroup’s livers showed marked vascular congestion, portal inflammation, and focal lytic necrosis (Fig. 5A). The livers of the cotrimoxazole-treated subgroup displayed significant deterioration, with marked hydropic degeneration of hepatocytes, multiple focal lytic necrosis, vascular congestion, and confluent necrosis (Fig. 5B). In contrast, the acute *n*-BuOH fraction-treated subgroup’s liver tissues appeared nearly normal, showing no congestion, inflammation, necrosis, or degeneration (Fig. 5C). The chronic non-treated group’s livers exhibited marked vascular congestion, portal mononuclear inflammatory infiltrate, focal lytic necrosis, and significant hydropic degeneration of hepatocytes (Fig. 5D). The livers of the chronic cotrimoxazole-treated group showed less vascular congestion, the presence of giant cells (indicating repair), and prominent focal lytic necrosis (Fig. 5E). Finally, the liver tissues of the chronic *n*-BuOH fraction-treated subgroup displayed mild vascular congestion, with no evidence of portal inflammation or focal lytic necrosis, and indications of repair, including binucleated hepatocytes (Fig. 5F).

#### NO, INF-γ, and TNF α levels in serum

**Fig. 6:**
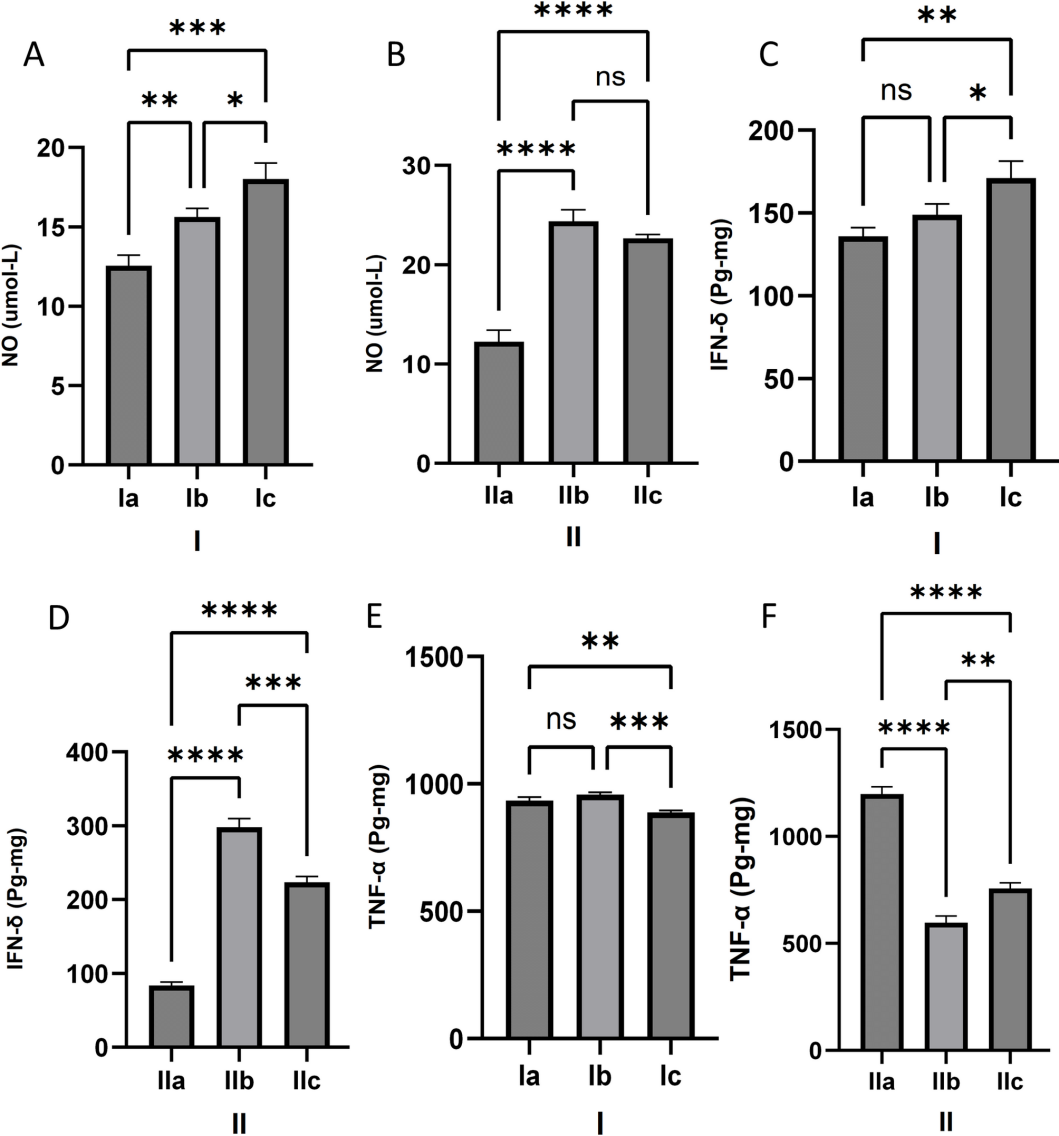
The serum levels of NO (**A** and **B**), IFN-γ (**C** and **D**), and TNF α (E and F) for experimental subgroups. Results are expressed as mean ± standard deviation and differences were considered significant at *p* < 0.05 (*), *p* < 0.01 (**), *p* < 0.001 (***), and *p* < 0.0001 (****) or non-significant (ns). The first group I: was orally inoculated with 20 cysts per mouse, and the second group II: was orally inoculated with 10 cysts. Ia, IIa: infected non-treated mice (control group), administrated 1% DMSO-PBS only (vehicle); Ib, IIb: infected and treated with the control drug, cotrimoxazole, at a dose of 370 mg/kg/day; and Ic, IIc: infected and treated with the tested plant extract (*C. rumphii n*-BuOH fraction) at a dose of 200 mg/kg/day.

The levels of nitric oxide (NO) in the serum of both the *C. rumphii n*-BuOH fraction and cotrimoxazole-treated subgroups were significantly higher than those in the non-treated infected groups, both acute and chronic (Fig. 6A **and B**). Notably, the drug-treated subgroup (Ic) displayed a significantly higher level of NO (18.01 ± 1.0 µmol/L) compared to Ia (*p* < 0.001), and Ib (*p* < 0.05) subgroups in the acutely infected subgroup. While it showed a significantly higher level when compared to non-treated mice in the chronically infected subgroup (22.65 ± 0.40 µmol/L) (*p* < 0.0001), but a non-significantly lower level when compared to the cotrimoxazole-treated subgroups (24.37 ± 1.15 µmol/L). Regarding the measured pro-inflammatory cytokines, the acutely infected subgroup treated with *C. rumphii n*-BuOH fraction exhibited significantly higher serum levels of IFN-γ (171 ± 10.19 pg/mg) compared to non-treated (*p* < 0.01). In contrast, the non-treated and cotrimoxazole-treated subgroups (Ia and Ib) had lower, non-significant levels of IFN-γ (135.9 ± 5.24 pg/mg, and 149 ± 6.54 pg/mg, respectively) (Fig. 6C). On the other hand, the cotrimoxazole-treated chronic subgroup demonstrated significantly higher serum IFN-γ levels (298 ± 11.80 pg/mg), compared to the other subgroups (Fig. 6D). The *C. rumphii n*-BuOH fraction-treated chronic subgroup also showed significantly elevated serum IFN-γ levels (223.4 ± 7.67 pg/mg) compared to the non-treated control subgroup (83.51 ± 4.78 pg/mg) (*p* < 0.0001). Nevertheless, the subgroup of acutely infected mice treated with *C. rumphii n*-BuOH fraction exhibited a significant reduction in serum levels of TNF-α (888.1 ± 7.95 pg/mg). This was notably lower than the levels observed in both the non-treated group (934.9 ± 12.75 pg/mg) and the cotrimoxazole-treated group (957.6 ± 8.76 pg/mg) (Fig. 6E). In the chronic model, the TNF-α levels in the non-treated group were significantly higher (1198 ± 33.81 pg/mg) compared to the other subgroups (*p* < 0.0001). However, the drug-treated subgroup still had elevated levels (756.5 ± 27.19 pg/mg), which were significantly higher than those in the cotrimoxazole-treated group (596.4 ± 31.50 pg/mg, *p* < 0.01) (Fig. 6F).

## Discussion

Murine models and humans have markedly different methods of innate immune detection and cytokine production to pathogen infections. Nonetheless, they remain an essential model for studying *T. gondii *infection and developing treatment strategies^38^. Effective therapeutic options for toxoplasmosis are currently limited, with no ideal treatment available due to their limited efficacy against the chronic stage of the infection and potential adverse effects^39^. The risks of side effects from pyrimethamine combinations highlight the need for alternative treatments. Trimethoprim-sulfamethoxazole (TMP-SMX) showed comparable efficacy on acute cerebral and ocular toxoplasmosis, while also being better tolerated^40–43^. However, TMP-SMX is also associated with a variety of adverse events, including rashes, allergic reactions, gastrointestinal discomfort, hyperkalemia, and signs of drug-induced liver injury^44,45^. Medicinal plant extracts and their natural metabolites have been shown to be promising alternative safe therapeutics for toxoplasmosis^18–20^. Extracts from *C. rumphii *have demonstrated antibacterial and anticancer properties^25–27^. However, the antiparasitic properties of various *Cycas* species have not been thoroughly investigated. The *n*-BuOH fraction of *C. rumphii* has shown promising potency against *T. gondii *(RH strain) in vitro when compared to the standard drug cotrimoxazole^27^. In the present study, we evaluated the efficacy in vivo on both the acute and chronic phases of *T. gondii* ME49 strain infection compared to TMP-SMX in experimental Swiss albino mice.

*Toxoplasma gondii *infection leads to a systemic infection that occurs in two stages: acute with spreading tachyzoites and chronic with encysted bradyzoites^46,47^. Several factors can influence the virulence in mice, including the parasite strain, method of infection, the number of parasite passages, and the specific mouse host line used^48^. Using less virulent strains in experimental mice enables effective drugs evaluation in both acute and chronic stages of infection^32,49^. The ME49 strain of *T. gondii *can lead to subclinical or chronic infections in mice. Inoculating 10 cysts typically results in lower mortality rates and variable cyst counts after four to eight weeks post-infection^49,50^. However, inoculating 20 cysts or more can lead to higher mortality rate^33,35,51^. Thus, this study was designated to evaluate the efficacy of the tested drug during the early phase (acute phase) of toxoplasmosis by initiating treatment on the 4 th day post infection with 20 cysts orally, and evaluate the efficacy on the brain cysts in the chronic or latent phase by starting therapy on the 6 th week post infection with 10 cysts orally. Oral administration of *C. rumphii n*-BuOH fraction, at a daily dose of 200 mg/kg for two weeks, significantly decreased the cyst count in the brains of infected mice, and improved the survival rate of treated mice, with results comparable to those observed in subgroups treated with cotrimoxazole in both acute and chronic models. The size of tissue cysts can also differ based on several factors, including the duration of the infection, the type of host cell, the strain of the parasite, and the method used for measurement. Tissue cysts can grow uniformly for the first ten weeks; however, after this period, there is significant variation in size. This variation may be attributed to the emergence of a second generation of tissue cysts^52^. The cysts from the *C. rumphii n*-BuOH fraction-treated animals were significantly smaller than those from the control non-treated subgroups in both acutely and chronically infected groups euthanized at eight and ten weeks, respectively.

HPLC analysis of the *n*-BuOH fraction of *C. rumphii* revealed that this fraction is rich in phenolic acids, including caffeic, gallic and chlorogenic acids. Only two flavonoids, (+)-catechin and naringenin, were detected in this fraction, and their concentrations were low. These findings align with the total phenolic content and total flavonoid content measurements, which indicated that the *n*-BuOH fraction contains more phenolic acids than flavonoids. The predominant phenolic acids identified in the *n*-BuOH fraction have been linked previously to antiparasitic and anti-toxoplasmosis properties. Caffeic acid, which is the most abundant phenolic acid in the *n*-BuOH fraction, along with its phenyl ester and amide derivatives, has been shown to act as specific inhibitors of nuclear factor-κB (NF-κB) and tubulin binders, both of which are essential for the survival of the intracellular parasite *T. gondii*^53,54^. Chlorogenic acid, another major phenolic acid identified in our fraction, has demonstrated activity against *T. gondii*, with an inhibition rate of 34.56% at a concentration of 400 µg/mL^55^. Furthermore, gallic acid has been evaluated for its anti-toxoplasmosis activity through an in-silico study targeting *T. gondii *thymidylate synthase and dihydrofolate reductase (TS-DHFR). The results indicated that gallic acid has a high binding affinity for inhibiting TS-DHFR^56^. Both caffeic and gallic acids have exhibited antiparasitic effects beyond *T. gondii*. Caffeic acid has shown efficacy against two different parasites, *Trypanosoma brucei rhodesiense* and *Leishmania donovani*. Similarly, gallic acid has demonstrated anti-trypanosomal activity against *T. brucei rhodesiense* and *T. cruzi*^57^. Furthermore, gallic acid displayed potent anti-schistosomal activity in mice infected with *Schistosoma mansoni*^58^. Additionally, catechin, the flavonoid detected in the extract, inhibited *T. brucei rhodesiense*^57^.

The histopathological findings indicated that *C. rumphii n*-BuOH fraction effectively alleviated brain and liver lesions caused by *T. gondii*. In contrast, subgroups treated with cotrimoxazole exhibited moderate brain gliosis and inflammation, with some areas still showing severe inflammatory responses. Moreover, the liver of the cotrimoxazole-treated subgroup displayed significant damage and necrosis. This differing inflammatory response may be attributed to the antioxidant and anti-inflammatory properties of the phenolic acids found in *C. rumphii n*-BuOH extract. Caffeic acid and its derivatives, such as caffeic acid phenyl ester, are recognized for their strong antioxidant and anti-inflammatory effects^59–63^. Similarly, gallic and chlorogenic acids also demonstrate potent antioxidant and anti-inflammatory activity^64–67^.

Elevated serum levels of NO and IFN-γ in *T. gondii*-infected subgroups treated with cotrimoxazole or *C. rumphii n*-BuOH fraction, compared to non-treated controls, highlight its immunomodulatory effects. Immune responses to *T. gondii *infection differ between the acute and chronic stages and are reliant on variations in phenotype, pathogenicity, and clinical consequences of the parasite strains^47,68,69^. Nitric oxide reduces *Toxoplasma *immunopathology and suppress tachyzoite proliferation in the brain, preventing toxoplasmic encephalitis^70,71^. The pro-inflammatory cytokine interleukin-12 (IL-12) stimulates the release of interferon-γ (IFN-γ) from CD4 + and CD8 + T cells, as well as natural killer (NK) cells during the early stage of infection. IFN-γ plays a key role in cell-mediated immunity against *T. gondii *by upregulating antimicrobial effector mechanisms^72–74^. In contrast, TNF-α levels were significantly lower in subgroups treated with *C. rumphii n*-BuOH and cotrimoxazole compared to non-treated groups in both acute and chronic models. These findings align with a study showing that quinoline-based compounds, including PPQ-8 and pyrimethamine/sulfadiazine regimens, significantly increase serum IFN-γ and NO while decreasing TNF-α levels^75^. The role of TNF-α in *T. gondii *infection remains controversial; however, it demonstrates a synergistic effect with IFN-γ in activating macrophages and inhibiting parasite replication. Nonetheless, mice lacking TNF-α signaling may be susceptible to chronic toxoplasmosis due to changes in IFN-γ expression during infection, rather than a deficiency in a specific effector mechanism that relies solely on TNF-α^76^.

## **Conclusions**

*C. rumphii n*-BuOH fraction significantly reduces the cyst count in the brains of infected mice, improves the survival rate, and alleviates brain and liver lesions induced by *T. gondii*. This fraction is rich in phenolic acids, which possess antioxidant, anti-inflammatory, antiparasitic, and anti-toxoplasmosis properties. Furthermore, the elevated serum levels of NO and IFN-γ observed in the *C. rumphii n*-BuOH treated subgroups may be attributed to the immunomodulatory effects of this fraction.

## Electronic supplementary material

Below is the link to the electronic supplementary material.

Supplementary Material 1

## Data Availability

All data generated or analysed during this study are included in this published article and its supplementary information files.
