# Supplementary material for: Phytochemical content of Cycas rumphii n-butanol fraction and antiprotozoal activity against Toxoplasma gondii in vivo
Source: Sci Rep. 2025 May 5;15:15697. doi: 10.1038/s41598-025-98993-y (PMC12053620; doi:10.1038/s41598-025-98993-y)

Phytochemical content of *Cycas rumphii n*-butanol fraction and antiprotozoal activity against *Toxoplasma gondii in vivo*

Hosam M. El-Seadawy[1], Amany E. Ragab[1], Mona El-Aasr[1], Kamilia A. Abo El-Seoud[1], Ayat A. Elblihy[2,3], El-Sayed El-Alfy[4]*, Hanan Abd Elgawad[2,3], Somaya Saleh[4], Heba Sheta[5], Rana Elseadawy[4]

[1] Department of Pharmacognosy, Faculty of Pharmacy, Tanta University, Tanta, 31527, Egypt.

[2] Department of Medical Parasitology, Faculty of Medicine, Mansoura University, Mansoura, 35516, Egypt.

[3] Program of medicine and surgery, Mansoura National University, Gamasa, 35516, Egypt.

[4] Parasitology Department, Faculty of Veterinary Medicine, Mansoura University, Mansoura, 35516, Egypt.

[5] Department of Pathology, Faculty of Medicine, Mansoura University, Mansoura, 35516, Egypt.

*** Corresponding author:** El-Sayed El-Alfy: sydnabil@mans.edu.eg

Table S1: Gallic acid concentrations and absorbance for calibration curve of TPC

Conc. (μg/mL)	Absorbance
187.5	0.59
250	0.76
375	1.14
500	1.58
750	2.28
1000	3.01

Table S2: Rutin concentrations and absorbance for calibration curve of TFC

Conc. (μg/mL)	Absorbance
31.25	0.029
62.5	0.054
125	0.103
250	0.186
500	0.363
1000	0.676

Figure S1. Calibration curves of TPC and TFC. **A)** Total phenolic calibration curve of gallic acid standard. **B)** Total flavonoid calibration curve of rutin standard.

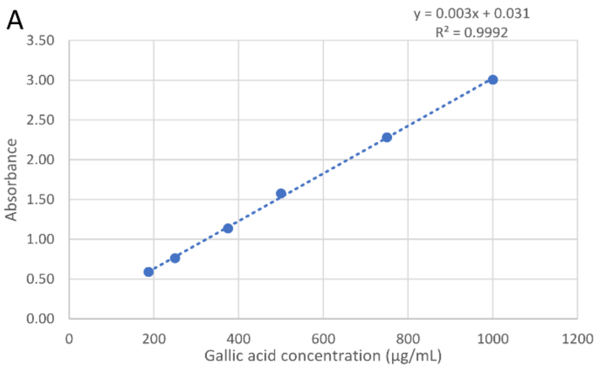

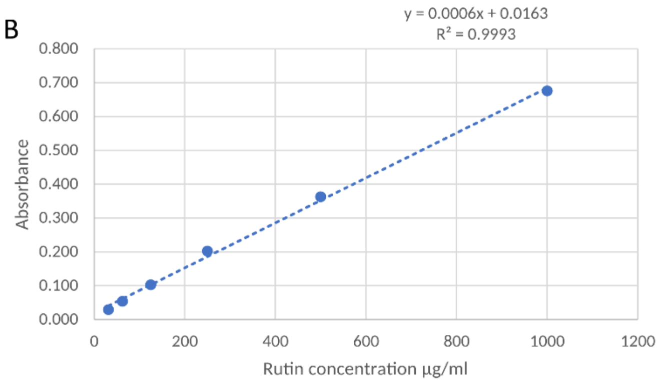

Supplement: Supplementary file 1 — Supplementary Material 1 [file 41598_2025_98993_MOESM1_ESM.pdf]
